# Increasing angiogenic efficacy of conditioned medium using light stimulation of human adipose-derived stem cells

**DOI:** 10.1038/s42003-022-03838-3

**Published:** 2022-09-13

**Authors:** Yu-Jin Kim, Sang Ho Lee, Jisoo Im, Jihun Song, Han Young Kim, Suk Ho Bhang

**Affiliations:** 1 School of Chemical Engineering, Sungkyunkwan University, Suwon, 16419 Republic of Korea; 2 Department of Biomedical-Chemical Engineering, The Catholic University of Korea, Bucheon, 14662 Gyeonggi Republic of Korea

**Keywords:** Stem-cell research, Regeneration

## Abstract

Conditioned medium (CM) contains various therapeutic molecules produced by cells. However, the low concentration of therapeutic molecules in CM is a major challenge for successful tissue regeneration. Here, we aim to develop a CM enriched in angiogenic paracrine factors for the treatment of ischemic diseases. Combining spheroidal culture and light irradiation significantly upregulates the angiogenic factor expression in human adipose-derived stem cells (hADSCs). Spheroids of light-irradiated hADSCs (SR group) show significantly enhanced expression of angiogenic paracrine factors compared with spheroids without light stimulation. Enhanced viability, migration, and angiogenesis are observed in cells treated with CM derived from the SR group. Furthermore, we performed in vivo experiments using a mouse hindlimb ischemia model; the results demonstrate that CM derived from densely cultured spheroids of light-irradiated hADSCs induced increased angiogenesis in vivo. In conclusion, our proposed approach of using light to stimulate stem cells may overcome the major drawbacks of CM-based therapies.

## Abstract (web summary)

Combining spheroidal culture of human adipose-derived stem cells with light irradiation enhances angiogenic growth factor secretion in conditioned media, which can improve angiogenesis in a mouse hindlimb ischemia model.

## Introduction

The possibility of treating ischemic disease using adult stem cells has been demonstrated in several studies^1,2^. It has been reported that the therapeutic effect of adult stem cells is exerted primarily because of paracrine factors, such as angiogenic paracrine factors, secreted by these cells^3–5^. Conditioned medium (CM) has attracted considerable attention as a therapeutic agent, given that various paracrine factors secreted by cultured cells are present in CM^4,5^. Paracrine factors present in CM can stimulate and treat target cells; therefore, CM administration is a potential therapeutic alternative to the direct injection of cells into the body. Paracrine factors released in stem cell CM are difficult to reproduce artificially^4,6^. Moreover, because CM is easy to store, it has immense potential as a therapeutic agent^7^.

For CM to be effective in clinical treatments, it is necessary to increase the concentration of the specific growth factors that are present in it^8,9^. In a clinical study in which human adipose-derived stem cells (hADSCs) CM were used to treat skin wounds, the skin rejuvenating effect of CM was shown; however, the skin tightening effect was not improved. The results of the study suggested that an insufficient amount of cytokines present in the CM was the cause of the poor therapeutic outcomes^10^. Although protein concentrators (e.g., centrifugal filters) are widely used to increase the concentration of growth factors in CM, they may alter the proportion of various molecules, and may not specifically increase the concentration of the growth factor crucial for the treatment of a particular disease, such as ischemic diseases^11,12^. Thus, priming cells to increase the concentration of specific paracrine factors in the CM is a promising strategy^13–15^. Furthermore, exogenous substances can be used to stimulate cells to secrete specific paracrine factors. After pre-stimulating mesenchymal stromal cells with TGF-β1 and lithium chloride, CM from these cells was found to contain increased amounts of inhibitory proteins and vascular endothelial growth factor (VEGF), and successfully improved hair growth in clinical trials^15^.

**Fig. 1:**
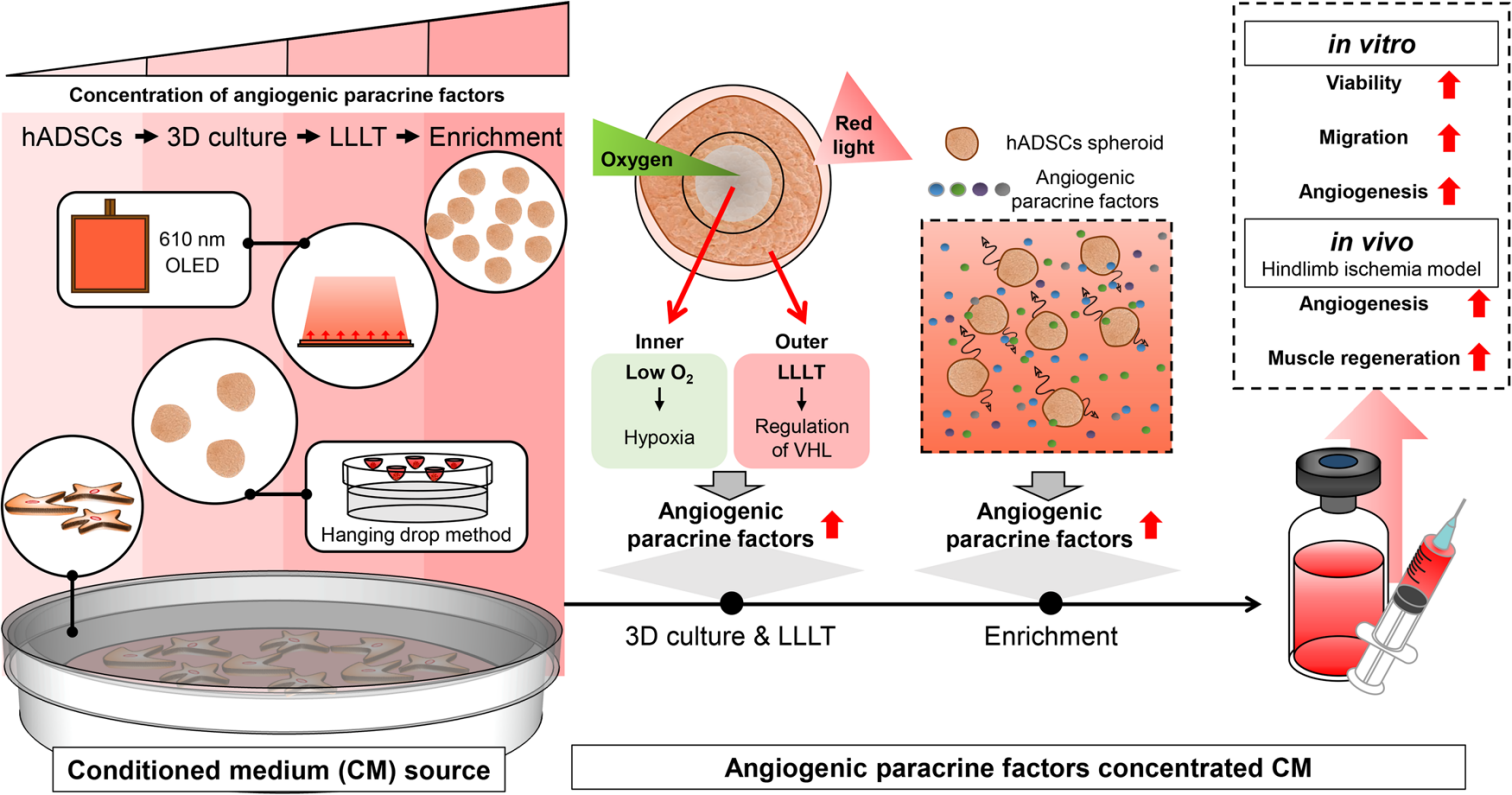
Schematic illustration showing the production of conditioned medium (CM) for ischemic disease therapy. *Left, Center*: Preparation of CM enriched with ischemic disease targeting-angiogenic paracrine factors obtained from human adipose-derived stem cells (hADSCs). *Right*: Positive effects relevant to the treatment of ischemic disease are listed.

In this study, we aimed to increase the concentration in CM of pro-angiogenic paracrine factors, which play major therapeutic roles in ischemic diseases^16,17^, by inducing cellular function, without the use of post-condensed processes or xenobiotics. The mechanistic process of concentrating angiogenic paracrine factors in CM (with the goal of treating ischemic diseases) is depicted in Fig. 1. CM was collected from hADSCs which are easily isolated and secrete various paracrine factors^18–20^. In addition, hADSCs were developed as three-dimensional (3D) spheroids and subjected to low-level light therapy (LLLT) to produce CM enriched in angiogenic paracrine factors.

It has been reported that hADSCs cultured as 3D spheroids secrete increased amounts of angiogenic growth factors compared to cells cultured in two-dimension (2D) culture^21,22^. When cultured as spheroids, the cells inside the spheroid are subjected hypoxia, which stimulates the secretion of growth factors^23–25^. However, if the spheroids contain a large number of cells, the cells inside the spheroid undergo necrosis, which affects spheroid viability^26,27^. In the present study, LLLT applied to hADSC spheroids utilizing organic light-emitting diodes (OLEDs) with a wavelength of 600–700 nm; this wavelength is primarily used for treating ischemic disease models using the conventional LLLT technique^28–30^. With existing techniques, the cells in the target lesion are mostly light-stimulated; therefore, it is difficult to establish a treatment protocol for LLLT in the clinical setting^31,32^. By combining these methods, we cultured a large number of hADSCs using the same volume of culture medium and applied LLLT to increase the secretion of growth factors. Importantly, in hADSCs grown as 3D spheroids, the internal cells are stimulated by the hypoxic environment, whereas the external cells are stimulated by LLLT, which significantly increases the intrinsic paracrine factor secretion capacity of hADSCs, without the intervention of exogenous substances. In summary, the approach used in this study improved the ability of hADSCs to secrete angiogenic paracrine factors by directly irradiating densely-populated hADSC spheroids with light (600–700 nm) under serum-free conditions. We evaluated the therapeutic effect of CM on hADSCs and human umbilical vein endothelial cells (HUVECs) cultured in vitro. Furthermore, the therapeutic ability of CM was evaluated in a mouse model of hindlimb ischemia. Our study provides strong evidence supporting the clinical application of CM in the treatment of ischemic diseases.

## Results

### Effect of red light treatment of hADSC spheroids on the secretion of angiogenic paracrine factors, appearance, and cell viability

**Fig. 2:**
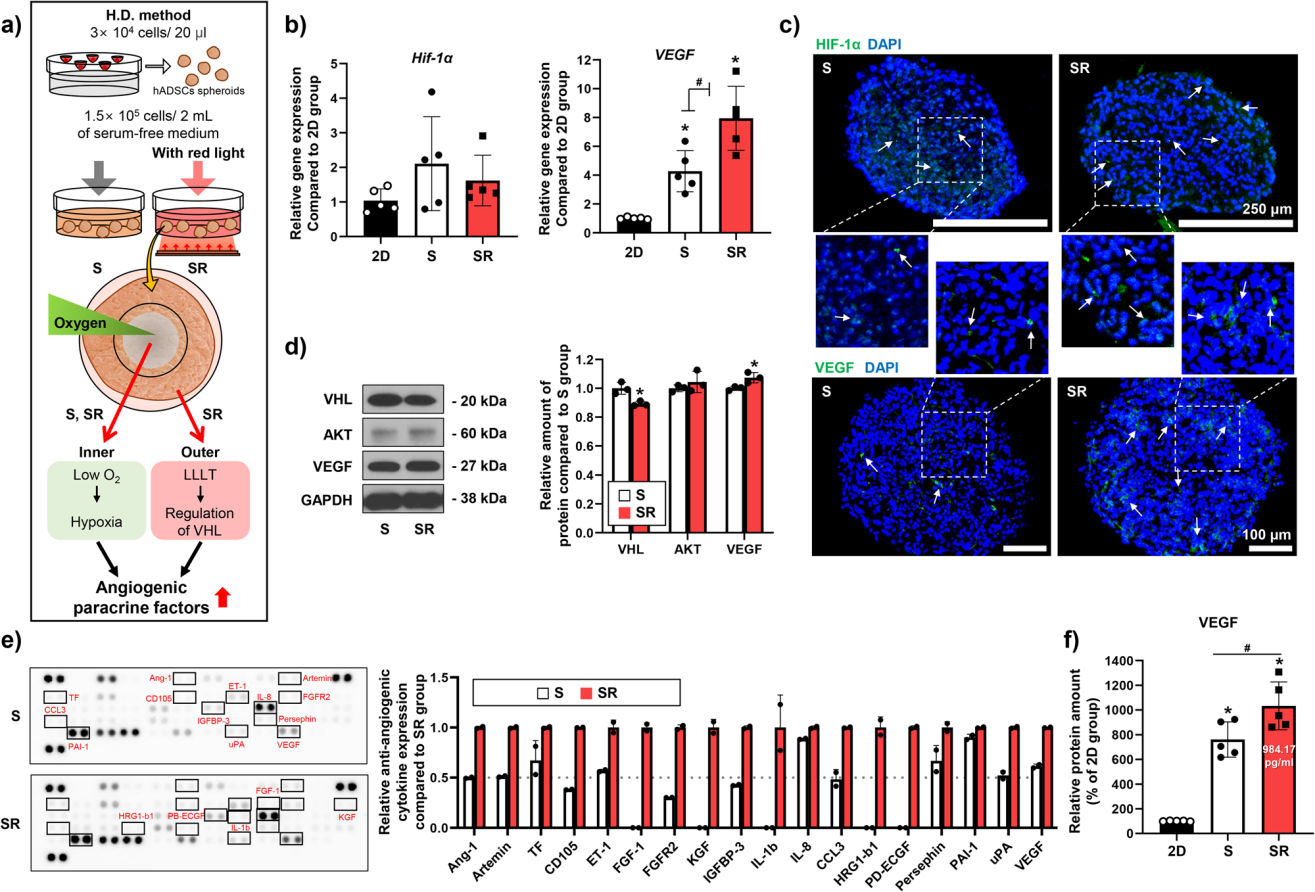
Secretion of angiogenic paracrine factors of spheroid hADSCs cells following red light stimulation. **a** Schematic depicting the three-dimensional culture of hADSCs and red light irradiation. **b** Relative expression of *HIF-1α* and *VEGF* in hADSC spheroids treated with red light (*n* = 5, *p < 0.05 vs. 2D group, #*p* < 0.05 vs. each group). **c** Representative images of HIF-1α and VEGF staining in the hADSC spheroids (green, HIF-1α or VEGF; blue, nuclei). HIF-1α (scale bar, 250 um) and VEGF (scale bar, 100 um) are indicated with white arrows. **d** Western blot analyses of key pro-angiogenesis paracrine molecules in hADSC spheroids treated with red light. Quantification of the relative protein level (*n* = 3, *p < 0.05 vs. S group). **e** Expression of angiogenesis-related proteins in CM, analyzed using human angiogenesis array. **f** Concentration of VEGF in the CM derived from 2D culture, S, or SR, as evaluated using enzyme-linked immunosorbent assay (ELISA; *n* = 5, *p < 0.05 vs. 2D group, #*p* < 0.05 vs. each group). Data are presented as mean ± S.D.

We present in Fig. 2a an outline of the experimental methods used in this study. When stem cells are cultured in a 3D spheroid form, their therapeutic efficacy is higher than that of cells grown in 2D culture^21,22^. One of the reasons for this phenomenon is that the cells experience hypoxia when cultured in the 3D form^23–25^. However, we confirmed that the expression of *HIF-1α*, which increases in the hypoxic cells, was not significantly different (Fig. 2b). One reason for this result may be that the average gene expression in hADSCs was deduced from the whole spheroid. In contrast, the expression of *VEGF*, which is upregulated by HIF-1α^33^, significantly increased in the 3D cultured group compared with that in the 2D group; furthermore, it increased markedly in the SR group (Fig. 2b). Subsequently, we assessed the degree of expression of HIF-1α and VEGF in the hADSC spheroids using immunohistochemistry (IHC) staining (Fig. 2c). HIF-1α and VEGF were concentrated inside the spheroids in the S group and throughout the spheroids in the SR group. Accordingly, we analyzed the expression of Von Hippel Lindau (VHL), AKT, and VEGF using western blotting. As shown in Fig. 2d, the expression of VHL was reduced in the SR group. VHL reduces HIF-1α stability by inducing hydroxylation of HIF-1α^34,35^. AKT expression was comparable between the two groups, but VEGF expression increased in the SR group. Additionally, by treating the spheroids with red light, the expression of VEGF in the hADSCs constituting the spheroids increased (Fig. 2b–d). Based on this, the collected CM was analyzed using an angiogenesis-specific array (Fig. 2e) and ELISA (Fig. 2f) to determine whether the hADSC spheroids released increased amounts of angiogenesis-related paracrine factors following red light treatment. The results showed that the expression of various factors was higher in the CM from the SR group than in the CM from the S group (Fig. 2e). The factors that can induce angiogenesis, namely FGF-1, KGF, IL-1b, HRG1-b1, and PD-ECGF, were detected only in the SR group, albeit in small amounts. The levels of pro-angiogenesis factors, namely TF, ET-1, IL-8, persephin, and Pal-1, were modestly higher in the SR group than in the S group. In particular, the levels of important pro-angiogenesis factors, namely Ang-1, artemin, CD105, FGFR2, IGFBP-3, CCL3, and μPA increased two-fold or more in the SR group than in the S group. Ang-1 can enhance angiogenesis in accordance with VEGF^36^. IGFBP-3 is also known to upregulate VEGF expression^37^. Subsequently, the concentration of VEGF in the CM (3 × 10^4^ cells/400 μL serum-free media) was quantified using ELISA; the VEGF concentration in the SR group was 984.17 pg·mL^-1^, which was 1.4-fold and 10-fold higher than the level found in the S group and 2D group, respectively (Fig. 2f).

**Fig. 3:**
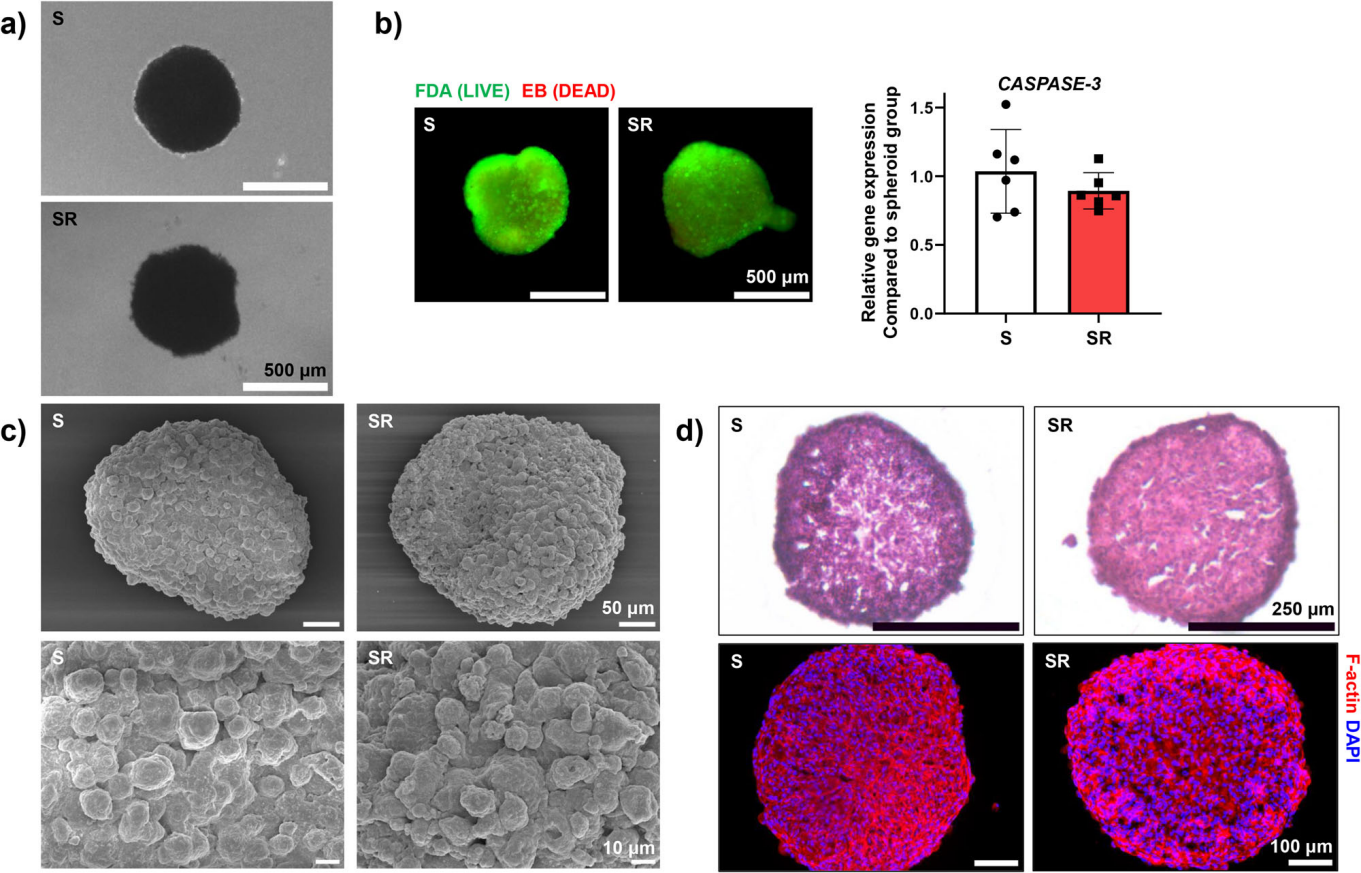
Spheroid morphology, and viability of spheroid hADSCs cells following red light stimulation. **a** Representative morphology of hADSCs spheroid following red light treatment. **b** Cellular viability of the hADSC spheroids following red light treatment, evaluated using the fluorescein diacetate ethidium bromide (FDA-EB) assay (green, live cells; red, dead cells; scale bars, 500 μm) and expression of *CASPASE-3* in hADSC spheroids (*n* = 6, Data are presented as mean ± S.D.). **c** Representative SEM image of fabricated spheroids. **d** H&E and F-actin staining in the interior of spheroids (S and SR).

The optical microscope images of the spheroids showed that there was no difference between the spheroid (S) group and the group of spheroids under red light treatment (SR) (Fig. 3a). Additionally, the fluorescent staining images of the live/dead cells indicated no significant differences in the number of dead cells (red) between the S and SR groups (Fig. 3b). In addition, there was no difference in the expression of *CASPASE-3* in hADSCs from the SR group compared to those from the S group (Fig. 3b). Furthermore, we examined whether there were any significant morphological changes in the spheroids after red light irradiation. To investigate the hADSC spheroid surface in more detail, scanning electron microscopy (SEM) was performed (Fig. 3c). The SEM images revealed no noticeable change in the morphology of the spheroids after red light treatment. In addition, no difference was observed in the internal structure of the spheroid sections (10 μm) after hematoxylin and eosin (H&E) and F-actin staining (Fig. 3d). When these results are considered together, we can confirm that red light treatment of hADSC spheroids did not change the spheroid structure or induce cytotoxicity.

### Enriching the angiogenic paracrine factor concentration in CM by stimulating hADSC spheroids with red light

**Fig. 4:**
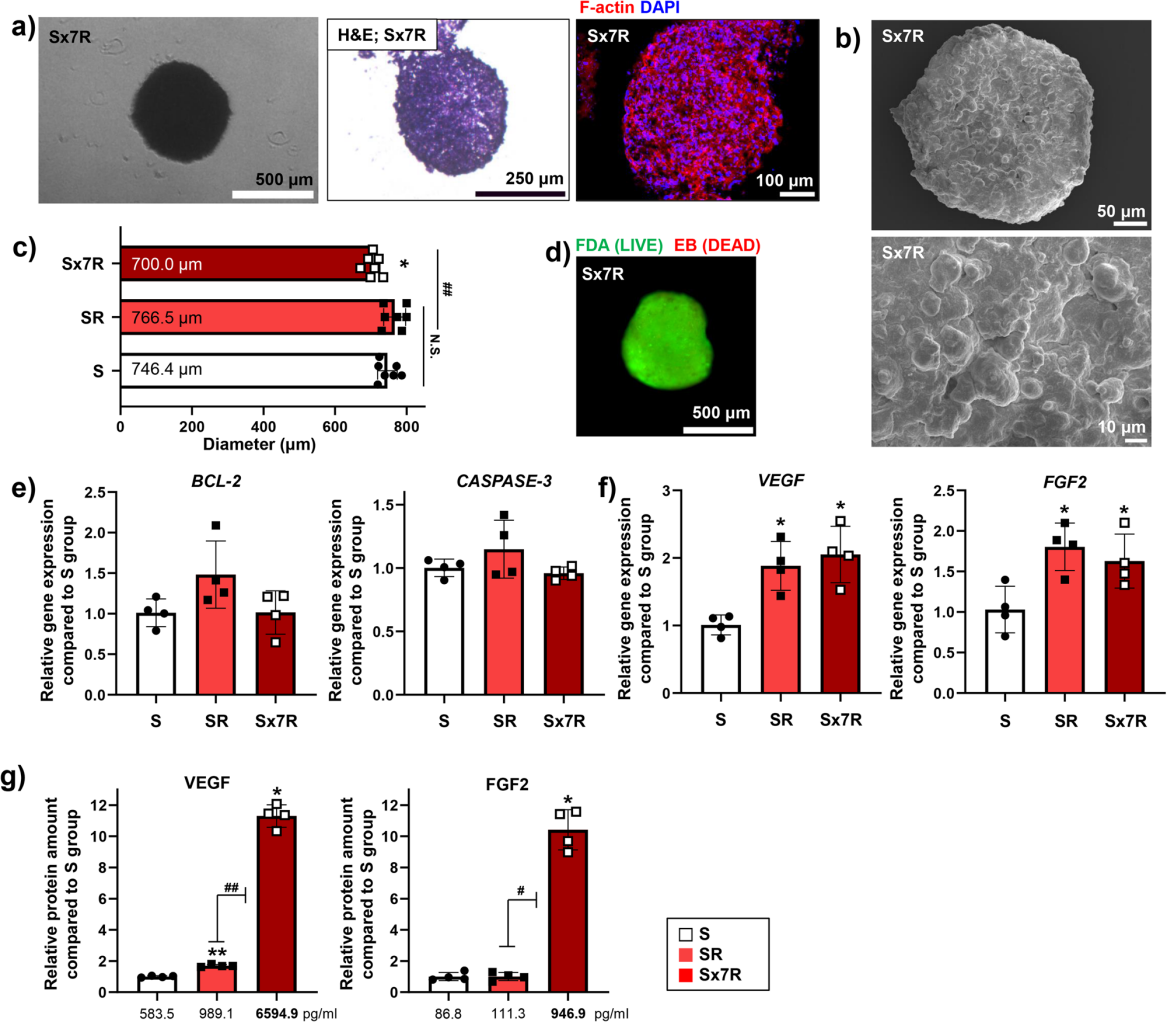
Structural properties, cell viability, and expression of angiogenesis-related paracrine factors in Sx7R. **a** Representative morphological features evaluated through H&E and F-actin staining of densely cultured hADSC spheroids treated with red light irradiation. **b** Representative SEM images showing the surface morphology of the spheroid. **c** Spheroid diameter measurements (*n* = 7, *p < 0.05 vs. S group, ##*p* < 0.001 vs. each group). The cell viability was evaluated using FDA-EB assay (**d**, scale bars = 500 μm) and the relative expression of *BCL-2* and *CASPASE-3* in hADSCs was analyzed using the S group as a control (**e**, *n* = 4). **f** Relative expression of *VEGF* and *FGF2* in the densely**-**incubated hADSCs spheroid following red light treatment using the S group as a control (*n* = 4, *p < 0.05 vs. the S group). **g** Relative concentration of VEGF and FGF2 in CM evaluated using ELISA (*n* = 5, *p < 0.05 vs. S group, **p < 0.001 vs. S group, #*p* < 0.05 vs. each group, ##*p* < 0.001 vs. each group). Data are presented as mean ± S.D.

3D culture enhances the number of cells that can be cultured per unit area compared with 2D culture. Thus, we attempted to increase the number of spheroids cultured in the same area and volume of serum-free medium. Concurrently, red light was used to treat the spheroids. Because light provides energy equally per spheroid, this technique allows us to apply the conditions used to cells in the previous SR group. According to previous ELISA results, the concentration of VEGF in the SR group was 984.17 pg·mL^−1^. Previous studies have shown that injecting or delivering 2.0–2.5 μg of VEGF to the ischemic region in mouse with 20 to 25 g body weight was effect to induce therapeutic angiogenesis^38,39^. On the other hand, studies applying CM, which contain various angiogenic paracrine factors, to the treatment of ischemic disease have shown a wide range of VEGF concentration^40,41^. According to a previous study, among CMs extracted from various cells, the treatment efficiency was best when the amount of VEGF delivered to mice through the CM was about 4 ng^40^. Therefore, if the concentration of VEGF observed in the SR group could be further enhanced by approximately seven times, the amount of VEGF sufficient for treatment could be delivered to the mouse lesion within 4 days via injection. First, spheroids were densely cultured (7x) in the same amount of serum-free medium and stimulated with red light (Sx7R group). The changes in spheroid structure and cytotoxicity were investigated. There were no significant changes in morphology (Fig. 4a–b); however, the diameter of the spheroids decreased to a certain extent (Fig. 4c). A decreased diameter was also observed when the same number of spheroids were cultured without light stimulation (Sx7 group, Supplemental Fig. 1a). Moreover, we observed no effect on cell viability in the live/dead assay (Fig. 4d) and expression of *BCL-2* and *CASPASE-3* (Fig. 4e) in the Sx7R group spheroids. A lack of cytotoxicity was also observed through the expression of apoptosis-related genes when the same number of spheroids was cultured without light stimulation (Supplemental Fig. 1b). Using quantitative reverse transcription polymerase chain reaction (RT-qPCR), we confirmed that light stimulation induced hADSC spheroids to enhance the expression of pro-angiogenic factors in the Sx7R group, as in the SR group (Fig. 4f). The expression of *VEGF* and *FGF2* was upregulated in the spheroids irradiated with red light, the SR and Sx7R groups, and had similar relative expression in both groups compared with that in the S group. When the number of spheroids was concentrated without exposure to light, the *VEGF* expression did not increase (Supplemental Fig. 1c). In addition, when the amount of VEGF and FGF2 was analyzed using ELISA, the Sx7R group had a markedly higher concentration, 6,594.9 pg·mL^−1^ for VEGF and 946.9 pg·mL^–1^ for FGF2, compared with that in other groups (Fig. 4g).

### Confirmation of the in vitro therapeutic efficacy of CM

**Fig. 5:**
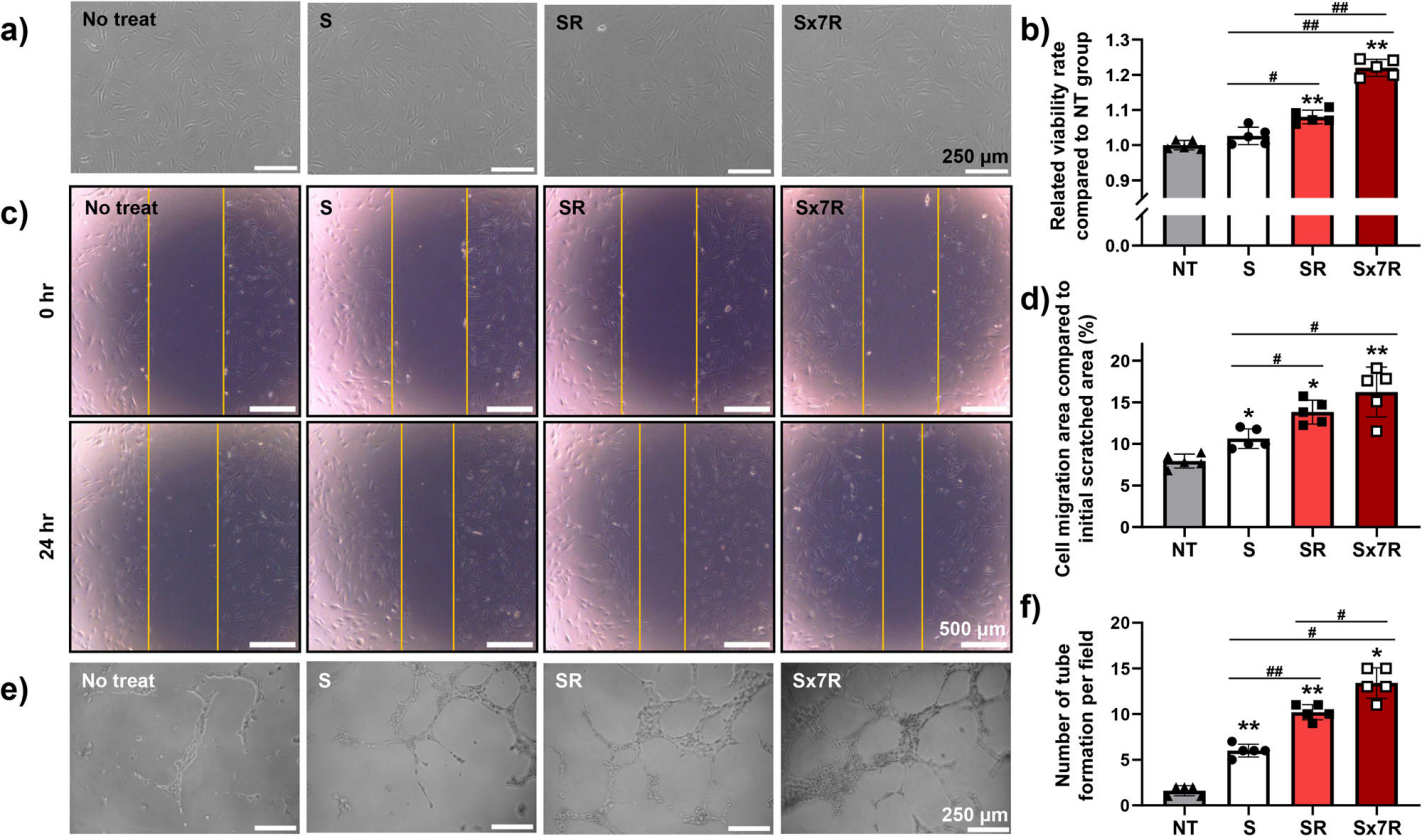
Angiogenic effect of conditioned medium (CM) collected from Sx7R in vitro. **a** Representative light microscopy images of hADSCs after the cells were treated with the various CMs for 24 h. **b** Relative cellular viability of hADSCs treated with the various CMs, evaluated using CCK-8 (*n* = 5, *p < 0.05 vs. no treatment (NT) group, **p < 0.001 vs. NT group, #*p* < 0.05 vs. each group, ##*p* < 0.001 vs. each group). **c**, **d** Representative image of scratch coverage in hADSCs after cells were treated with the various CMs for 24 h. The relative cell migration area compared with the initial scratch area is also shown (*n* = 5, *p < 0.05 vs. NT group, **p < 0.001 vs. NT group, #*p* < 0.05 vs. each group). **e**, **f** To determine the angiogenesis ability of different CMs on the HUVEC, the tube formation assay was performed to investigate HUVEC after the cells were treated with CM for 12 h (*n* = 5, *p < 0.05 vs. NT group, **p < 0.001 vs. NT group, #*p* < 0.05 vs. each group, ##*p* < 0.001 vs. each group). Data are presented as mean ± S.D.

The therapeutic potential of the CM collected after each culture method was confirmed in vitro. When hADSCs cultured in 2D were exposed to the various CMs for 24 h, no morphological differences were observed (Fig. 5a), whereas cell viability increased in the Sx7R group, followed by that in the SR group, compared with that in the other groups (Fig. 5b). However, the S group showed no difference in hADSCs viability compared to the no treatment (NT) group. To investigate the improvement in hADSC migration ability after treatment with different CMs, a scratch assay was performed (Fig. 5c, d). Twenty-four hours after scratching the hADSCs, maximum wound covering was observed in the Sx7R group. Lastly, we examined whether CM enriched with concentrated angiogenesis-related paracrine factors increased the angiogenic ability of endothelial cells (Fig. 5e, f). HUVECs exposed to CM for 12 h and all groups exposed to CM showed improved angiogenesis compared with that in the NT group, and maximum tube formation was observed in the Sx7R group.

### Verification of the therapeutic effect of CM in vivo

**Fig. 6:**
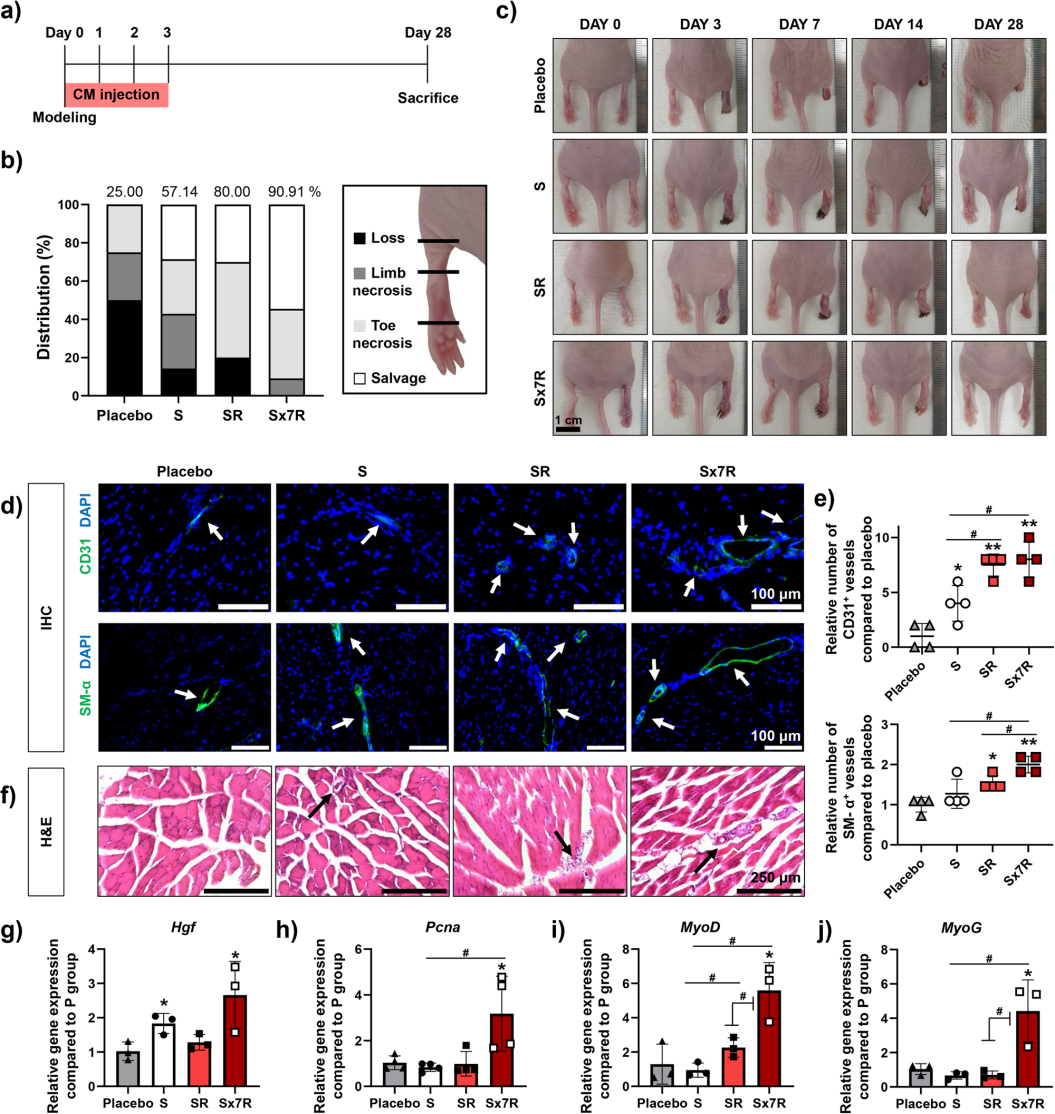
Treatment of hindlimb ischemic disease using conditioned medium (CM) collected from Sx7R. **a** Schedule of in vivo experiment. **b** Physiological status of hindlimb ischemia 28 days after the various treatments. **c** Representative photographs of limbs in each group at 0, 3, 7, 14, and 28 days after various treatments. **d** IHC images of CD31 or SM-α-actin expression (green fluorescence) and DAPI (blue) staining in the hindlimb regions on day 28 (Scale bar = 100 μm: white arrows indicate vessels). **e** Relative number of CD31^+^ and SM-α^+^ vessels in the hindlimb regions on day 28 (*n* = 4, *p < 0.05 vs. Placebo group, **p < 0.001 vs. Placebo group, #*p* < 0.05 vs. each group). **f** Representative images of H&E staining in the hindlimb regions on day 28 (Scale bar = 250 μm: black arrows indicate blood vessels). Relative expression of **g** *Hgh*, **h** *Pcna*, **i** *MyoD*, and **j** *MyoG* in the hindlimb regions on day 28 (*n* = 3, *p < 0.05 vs. Placebo group, #*p* < 0.05 vs. each group). Data are presented as mean ± S.D.

To confirm the therapeutic efficacy of CM for ischemic disease observed in vitro, ischemic hindlimb recovery was evaluated using a mouse ischemic hindlimb model^42,43^. The experimental protocol followed guidelines for the ethical treatment of experimental animals, as detailed in the Methods section below. The experimental schedule is shown in Fig. 6a. Each CM was injected into the gracilis muscle (150 μL·day^-1^) for 4 days, from 0 to 3 days after surgery. Mice were divided into the following four groups: PBS (placebo), CM collected from the S group (S), CM collected from the SR group (SR), and CM collected from the Sx7R group (Sx7R). Twenty-eight days after the various treatments, the mice from each group were classified in the order of limb salvage, toe necrosis, and limb necrosis, and loss, based on their limb conditions (Fig. 6b). The highest proportion of mice that recovered relatively well (limb salvage and toe necrosis) was 90.01% in the Sx7R group, followed by 80.00% in the SR group, and 57.14% in the S group, compared with that in the placebo group, with a percentage of only 25%. In particular, none of the mice in the Sx7R group experienced limb loss. We present in Fig. 6c representative photographs of the mice at 0, 3, 7, 14, and 28 days. On day 3, a difference in the treatment effect was clearly visible. On day 28, the number of microvessels in ischemic tissues was evaluated using IHC staining (Fig. 6d) and quantified (Fig. 6e). CD31^+^ microvessels were abundant in both the SR and Sx7R groups injected with CM collected from light-stimulated spheroids compared with that in the other groups, whereas SM-α actin^+^ microvessels were abundant in the Sx7R groups compared with that in the other groups. Laser Doppler Perfusion images showed improved blood flow in the Sx7R groups at day 3, 14, 21, and 28 compared with that in the S group (Supplemental Fig. 2, Supplemental Note 1). Additionally, the CM retrieved from densely-incubated hADSC spheroids stimulated with red light (Sx7R group) showed significantly improved tissue regeneration compared to that from the other groups, as evident from the H&E staining results (Fig. 6f). The expression in the ischemic limb of *Hgf* (Fig. 6g) and *Pcna* (Fig. 6h), both related to the promotion of regeneration and proliferation^44,45^, was quantified using RT-qPCR. The expression of *Hgf* was upregulated in the S and Sx7R groups compared to that in the placebo group. The expression of *Pcna* was upregulated in the Sx7R group compared with that in the placebo and S groups. Next, we analyzed the expression of *MyoD* (Fig. 6i) and *MyoG* (Fig. 6j), which are related to muscle regeneration and homeostasis^46,47^, in the ischemic limb, which was quantified using RT-qPCR. The SR group had higher *MyoD* expression than the S group, and higher expression was observed in the Sx7R group than in the SR group. In addition, the Sx7R group showed upregulated *MyoG* expression compared to the other groups. In summary, owing to continuous CM treatment during the initial 4 days, the CM from the Sx7R group, which had a very high concentration of pro-angiogenic paracrine factors, restored blood vessels faster than that in the other groups, and thus, the animals experienced decreased muscle loss.

## Discussion

In this study, we used 3D culture of hADSCs, red light treatment, and dense cell culture to produce CM with sufficiently high concentrations of pro-angiogenic paracrine factors for the treatment of ischemic disease. As detailed in the Results, the angiogenic ability of hADSCs cultured in 3D was significantly enhanced compared to that in the 2D culture^21,22^. To increase the concentration of pro-angiogenic paracrine factors in CM, hADSCs were cultured in a 3D spheroid form. In addition, red light was used to irradiate the spheroids during culture to induce CM production in a serum-free medium (Fig. 2a). Depending on the irradiation conditions, cells treated with red light induce the secretion of angiogenic paracrine factors via intercellular changes^28–30^. The red light used for stimulation in this study increased the secretion of angiogenic paracrine factors from the hADSC spheroids without causing any structural or morphological changes in the spheroid or a decrease in cell viability (Figs. 2–3). In particular, the results of IHC staining confirmed that HIF-1α and VEGF proteins were evenly produced inside and outside the spheroids in the SR group (Fig. 2e). A significant increase in *HIF-1α* expression was not observed (Fig. 2b). Nevertheless, because the expression of *VEGF* increased significantly in both the S and SR 3D culture groups compared to that in the 2D group (Fig. 2b), we inferred that the level of HIF-1α protein was stably maintained. In addition, the VEGF expression in the SR group was upregulated compared with that in the S group (Fig. 2b–d). We confirmed a decrease in VHL levels in the SR group (Fig. 2d). VHL expression is known to decrease under hypoxia^48,49^ and the SR group showed upregulation in intracellular ROS level after treatment compared to the S group (Supplemental Fig. 3a). In other words, unlike the S group, in which only the cells inside the spheroid were exposed to a hypoxic environment, the SR group showed differences in the level of VHL expression because the outer cells of the spheroid also experienced mild hypoxia due to light exposure. The hypoxic environment enhances AKT activation in cells^50^, but we did not observe any difference in AKT (Fig. 2d) and phospho-AKT expression (Supplemental Fig. 3b) between the S and SR groups. Therefore, the decreased level of VHL in the SR group may have increased the levels of angiogenic paracrine factors by enhancing HIF-1α stability. In addition, we analyzed angiogenic paracrine factors in the CM. By inducing intercellular changes in spheroids using red light, we confirmed the increased concentration and levels of various angiogenic paracrine factors in the CM (Fig. 2e). In particular, ELISA results showed that VEGF levels increased more than 10-fold in the same number of hADSCs compared with that in the 2D group (Fig. 2f). Additionally, we have found that expression of light-sensitive channels, such as *TRPV4*, was upregulated in the SR group compared with that in the S group (Supplemental Fig. 4a). *TRPV4* is known to enhance VEGF expression in cells^51–53^.

Furthermore, densely cultured hADSC spheroid cells with simultaneous red light stimulation secreted an increased amount of angiogenic paracrine factors in the CM. Using ELISA, we confirmed the high concentrations of VEGF and FGF2 in the CM of the Sx7R group. Compared to the S group, the secretion of VEGF and FGF2 increased by approximately 7-fold or more in the CM of the Sx7R group (Fig. 4g). These results were achieved by simply increasing the number of spheroids cultured in the same area and volume of medium when performing light treatment under the same conditions as the S group. If we increase the concentration of angiogenic paracrine factors through special drug or treatment with nanoparticles, we must also increase the number of those that enter in proportion to the number of cells. However, in this study, because the change in cells was induced by light, it was possible to obtain a relatively high concentration without changing the treatment conditions. We then investigated the therapeutic efficacy of the different CMs. Our highly concentrated CM (from the Sx7R group) effectively increased the viability and migration ability of hADSCs (Fig. 5a–d), and this may have been due to the high VEGF concentration in the CMs. In addition, the CM generated in our study contained a variety of angiogenic paracrine factors at high concentrations, effectively enhancing the angiogenic ability of HUVECs (Fig. 5e, f). Moreover, in the in vivo study (Fig. 6), the CM from the Sx7R group showed a better therapeutic effect; indeed, SM-α^+^ microvessels were abundant in the Sx7R group, and none of the mice in this treatment group experienced limb loss. The SR group also showed enhanced treatment efficiency compared with the S group, probably because the CM from the SR group contained factors with increased concentrations due to light stimulation. According to previous studies, it is reported that the therapeutic efficiency of ischemic disease increases when several factors were injected together rater than single factors such as VEGF or HGF. Many researchers are investigating the combination of factors that are effective when delivered together to enhance therapeutic efficacy. Moreover, when injecting several factors together, it showed an enhanced therapeutic effect even at a lower concentration of factors than single factors injection^36,54,55^. Therefore, we expect that the overall effect on ischemic disease treatment might be enhanced in Sx7R CM than in VEGF single injection, even concentration of VEGF in Sx7R CM was low. Notably, the expression of muscle regeneration-related genes improved in the ischemic limb in the Sx7R group (Fig. 6i, j). The Sx7R group, which had a very high concentration of pro-angiogenic paracrine factors, restored blood vessels faster than in other groups. As result, the model animals showed a decrement in muscle degeneration. Since *MyoD* and *MyoG* are myogenic regulators which paly important roles in regulating muscle differentiation^47,56^, the increased expression of myogenic genes might induce muscle differentiation and stimulate muscle growth that can potentially help muscle homeostasis.

Here, we developed hADSC spheroids that promoted angiogenic paracrine factor secretion in response to directly applied LLLT techniques. With this approach, we aimed to overcome the limitations of conventional CM and increase its treatment efficiency. To the best of our knowledge, there have been few studies focused on utilizing the LLLT technique for irradiating diseased areas and tissues and not the cells themselves. As the depth of diseased area and tissue increases, the transmittance of light to the tissue decreases rapidly. Because tissue is composed of several complex molecules, it is not entirely reasonable to expect the desired therapeutic effect on the diseased area with the current LLLT technique, which irradiates the tissue directly^57^. The current LLLT technique uses a laser or LED as a light source^58^; in contrast, we selected OLEDs because they generate less heat than do conventional light sources and have the advantage of being applied in wearable devices^59^.

In this study, hADSCs were cultured into a 3D spheroids to increase the concentration of angiogenic paracrine factors in the CM, and favorable results were obtained. When cells are cultured in 3D forms, they are exposed to a mildly hypoxic environment, and the secretion of angiogenic paracrine factors increases according to the interaction between the hypoxic environment and growth factors^23–25^. Although the expression of *HIF-1α* showed slightly different results than expected, this may be due to the short duration of *HIF-1α*^60^. For a more accurate analysis, it is necessary to optimize the time point at which *HIF-1α* expression changes^61^. When HIF-1α was inhibited using HIF-1α inhibitor, the level of VEGF in the CM derived from S or SR with HIF-1α inhibitor groups was markedly decreased when compared to the S or SR group (Supplemental Fig. 4b). Additionally, the gene expression of *TRPV4* in the SR with HIF-1α inhibitor group was markedly increased compared to the S with or without HIF-1α inhibitor groups (Supplemental Fig. 4c). Therefore, we have concluded that the upregulation of HIF-1α and *TRPV4* expression by light treatment enhanced the angiogenic paracrine factors. Because the CM with highly concentrated angiogenic paracrine factors developed in this study does not contain exogenous cells or chemical compounds, it is expected to have no adverse effects, such as an immune response, after injection and transplantation to the disease area.

We performed 3D culture and light treatment of hADSCs to increase the expression of angiogenic factors in the CM. Presumably, if we further investigated the changes in factor expression by hADSCs by modulating the light treatment conditions, our method could be used for the production of CMs for the treatment of other diseases. In other words, it will be possible to overcome the limitations of CM with a low concentration of therapeutic molecules and produce a therapeutic agent with highly concentrated paracrine factors to treat diseases without injecting drugs or exogenous substances such as nanoparticles. The CMs developed by our method are expected to show similar effectiveness as cell therapy without injecting cells into the disease area.

In this study, to develop a CM-based injection treatment containing highly concentrated angiogenic paracrine factors, hADSC spheroids were densely cultured in serum-free medium and treated with red light using OLEDs. First, we demonstrated that culture and treatment did not induce any internal or external changes in the morphology or cytotoxicity of the spheroids. The CM collected from hADSC spheroids stimulated with red light increased the types and concentrations of various angiogenic paracrine factors, and we showed that even in densely-incubated spheroids, it was possible to easily increase the cytokine concentration within CM while maintaining enhancement of angiogenic paracrine secretion through light stimulation. In addition, the therapeutic effects of CM have also been demonstrated. Cell viability, mobility, and angiogenesis were increased by applying them to in vitro cell culture. In vivo, mice injected with CM derived from Sx7R showed enhanced angiogenesis. Therefore, the CM developed in this study effectively increased and naturally concentrated angiogenic paracrine factors among cytokines secreted by hADSCs through 3D culture, light stimulation, and dense culture. Consequently, we expect that the technique we have demonstrated in this study will be highly useful for the treatment of ischemic disease in the future.

## Methods

### Cell culture

The hADSCs and HUVECs used in this study were purchased from Lonza (Walkersville, MD, USA). hADSCs were cultured in Dulbecco’s modified Eagle’s medium (DMEM, Gibco BRL, Gaithersburg, MD, USA) supplemented with 1% (v/v) penicillin (PS, Gibco BRL), and 10% (v/v) fetal bovine serum (Gibco BRL). HUVECs were cultured in endothelial cell growth medium-2 (EGM-2; Lonza). The culture medium was replenished every other day. hADSCs amd HUVECs with fewer than six passages were used for the experiments.

### Isolation of CM

For 2D culture, hADSCs were seeded on 6-well culture plates (1.2 × 10^5^ cells/well). One day after seeding, the cells were washed with PBS and then incubated with 2 mL serum-free DMEM (Gibco BRL), supplemented with 1% (v/v) PS (Gibco BRL) for 24 h. For 3D culture, the hanging drop method was used. Briefly, droplets (3 × 10^4^ hADSCs in 20 μL medium per droplet) were deposited on the lids of 100-mm culture dishes and the lids were inverted. The dishes were then filled with 10 mL PBS (Gibco BRL) to prevent drying. After 24 h of incubation (5% CO_2_ incubator at 37 °C), the spheroids were collected from the lids and washed with PBS (Gibco BRL). To collect CM from the hADSC spheroids produced by the handing**-**drop method, the fabricated spheroids were cultured in serum-free medium (serum-free DMEM (Gibco BRL) supplemented with 1% (v/v) PS (Gibco BRL)) for 24 h on a non-adhesion plate. Five or 35 spheroids were loaded per well in 6-well plates pre**-**coated with 2% (w/v) poly(2-hydroxyethyl methacrylate) (Sigma-Aldrich, St. Louis, MO, USA) in 95% ethanol and sterilized on a clean bench overnight using UV light. The spheroids were incubated with 2 mL serum-free medium (spheroids; S group). By irradiating the spheroids with red light, we aimed to enhance the angiogenic paracrine factor secretion ability of the spheroid and increase the concentration of angiogenic paracrine factors in CM (spheroids following red light treatment; SR or Sx7R group)^62^. After each spheroid group was incubated for 24 h with 2 mL serum-free DMEM, the culture medium was collected from each well and passed through a 0.1-μm polyvinylidene fluoride filter (Millipore, Burlington, MA, USA) to remove cells and debris. The CM was stored at -80 °C until further use.

### Quantitative real-time polymerase chain reaction (qPCR)

Total RNA was extracted from the samples using TRIzol (Ambion, Austin, TX, USA) according to the manufacturer’s protocol and reverse-transcribed into cDNA using Primescript RT Master Mix (Takara, Kusatsu, Japan), followed by RT-qPCR amplification using SsoAdvanced Universal SYBR Green Supermix (Bio-Rad, Hercules, CA, USA) and a CFX Connect™ real-time PCR detection system (Bio-Rad). For the in vitro assay, RT-qPCR was performed to quantify the relative expression of *CASPASE-3*, *VEGF*, *HIF-1α*, B-cell lymphoma 2 (*BCL-2*), and fibroblast growth factor 2 (*FGF2*). Glyceraldehyde 3-phosphate dehydrogenase (*GAPDH*) was used as an internal control. For the in vivo assay, total RNA was extracted from the retrieved ischemic limb tissue and RT-qPCR was performed to quantify the relative expression of hepatocyte growth factor (*Hgf*), proliferating cell nuclear antigen (*Pcna*), *MyoD*, and *MyoG*. *Beta-actin* (*Actb*) served as the internal control. Primer sequences are listed in Supplementary Table 1.

### Spheroid characterization

After treatment, the spheroids were fixed with 4% paraformaldehyde (Biosesang, Sungnam, Korea) for 10 min at room temperature. Spheroid morphology was analyzed using a microscope (CKX53, Olympus, Tokyo, Japan) and a scanning electron microscope (JSM-6510, JEOL, Tokyo, Japan). The fixed spheroids were embedded in optimal cutting temperature (OCT) compound (Scigen Scientific, Gardena, CA, USA). After the freezing step, the samples were cut into 10 μm sections at -20 °C, and sections containing spheroids were stained with H&E and phalloidin (F-actin) to assess the inner structure of the spheroids. For phalloidin staining, sections were stained with TRITC-phalloidin containing a mounting medium (VECTASHIELD H-1600, Vector, Burlingame, CA, USA) and 4ʹ,6-diamidino-2-phenylindole (DAPI, Vector). In addition, immunofluorescence staining was performed to visualize hypoxia-inducible factor-1α (HIF-1α) and VEGF expression using anti-HIF-1α (1:500, Abcam, Cambridge, MA, USA, ab179483) and anti-VEGF (1:100, Abcam, ab183100) antibodies and a fluorescein isothiocyanate-conjugated secondary antibody (1:50, Jackson ImmunoResearch Laboratories, West Grove, PA, USA, 11, 111-095-144). The sections were stained with DAPI and examined under a fluorescence microscope (DFC 3000 G; Leica, Wetzlar, Germany).

Protein was extracted from the samples using a radioimmunoprecipitation assay buffer (Sigma-Aldrich). To quantify protein concentration, the bicinchoninic acid assay (ThermoFisher Scientific, Lenexa, KS, USA) was conducted. Proteins were boiled at 100 °C for 5 min in 4× Laemmli sample buffer (Bio-Rad) containing β-mercaptoethanol, and the equivalent amounts of protein were loaded onto a 10 % SDS-PAGE gel. We transferred separated proteins onto immune-blot polyvinylidene fluoride membranes. The membranes were blocked with 1× TBS-T (Bio-Rad), containing 5 % skim milk, for 1 h at room temperature, then were incubated overnight with primary antibodies. We washed the membranes with 1× TBS-T and incubated them with secondary antibodies for 1 h at room temperature. Following TBS-T washes, protein bands were visualized using the ECL reagent WESTSAVE UP (ABfrontier, Seoul, Korea), and the membranes were exposed to X-ray films. To analyze the expression of bands, the Image J software (National Institutes of Health, Bethesda, MD, USA) was used. The following antibodies were used for western blot analysis: anti-VHL (1:1000, Cell Signaling Technology, Danvers, MA, USA, CS3285), anti-AKT (1:1000, Cell Signaling Technology, CS4691), anti-P-AKT (1:500, Cell Signaling Technology, CS4060), anti-VEGF (1:1000, Abcam, ab46154), and anti-GAPDH (1:2500, Abcam, ab9485), GAPDH was used as internal controls.

### CM analysis

The amounts of VEGF and FGF2 in CM were quantified using human VEGF DuoSet ELISA (R&D Systems, Minneapolis, MN, USA) and human FGF basic/FGF2/bFGF DuoSet ELISA (R&D Systems), following the manufacturer’s instructions. Optical density was measured using a microplate reader (450 nm, correction 540 nm, Infinite F50, Tecan, Mannedorf, Switzerland). The Proteome Profiler Human Angiogenesis Array Kit (R&D Systems) was used to analyze the expression of pro-angiogenic proteins in CM according to the manufacturer’s protocol. The pixel density of each spot was quantified using Image J software (National Institutes of Health), and the average signal was calculated for duplicate spots.

### Fluorescein diacetate/ethidium bromide (FDA/EB) assay

An FDA/EB assay was performed to evaluate cell viability. FDA (green, Sigma-Aldrich) stains the cytoplasm of viable cells, whereas EB (red, Sigma-Aldrich) stains the nuclei of non-viable cells. The staining solution was freshly prepared by mixing 10 mL FDA stock solution (1.5 mg·mL^−1^ FDA in dimethyl sulfoxide), 5 mL EB stock solution (1 mg·mL^−1^ EB in PBS), and 3 mL PBS. The cells were then incubated with the staining solution for 3–5 min at 37 °C. After staining, the samples were washed two to three times with PBS and examined under a fluorescence microscope (DFC 3000 G).

### Cell viability and cell migration ability

Cell viability was analyzed using the Cell Counting kit-8 (CCK-8; Dojindo Molecular Technologies, Inc., Kumamoto, Japan). hADSCs were seeded on 24-well plates (1.5 × 10^4^ cells/well) and incubated with CM for 24 h. Subsequently, the cells were washed with PBS and incubated with 10% (v/v) CCK-8 solution for 3 h at 37 °C. The optical density was measured at 450 nm (Infinite F50). Cell migration ability was assessed using a scratch assay. Briefly, hADSCs were cultured in 24-well plates until they reached confluency. The cell monolayer was scratched using an SPLScar™ Scarther 24 well (SPL Life Sciences, Pocheon, Korea) to create a linear gap. Floating cells were removed by washing with PBS, and CM was added to each well. Cell migration was examined under a microscope (CKX53) for 24 h. The relative migration area was expressed as: [(scratched area – remaining area) / scratched area]  × 100.

### Endothelial cell tube formation assay

An angiogenesis assay kit (in vitro) (ab204726, Abcam) was used to perform the endothelial cell tube formation assay, in accordance with the manufacturer’s instructions. Briefly, HUVECs were seeded onto an extracellular matrix gel (2 × 10^4^ cells per well) in 100 μL sample medium and incubated for 12 h at 37 °C. Following incubation, the HUVECs were observed under a microscope (CKX53).

### Hindlimb ischemia models

A mouse hindlimb ischemia model was established as previously described^42,43^. Four-week-old female athymic mice (20–25 g body weight; Orient Bio Inc., Sungnam, Korea) were anesthetized with an intraperitoneal injection of xylazine (10 mg·mL^−1^) and ketamine (100 mg·mL^−1^). The femoral artery and its branches were ligated via a skin incision using a 6–0 silk suture (Ethicon, Somerville, NJ, USA), along with the external iliac artery and all upstream arteries. The femoral artery was excised from its proximal origin as a branch of the external iliac artery to the distal point, where it bifurcated into the saphenous and popliteal arteries. Immediately after arterial dissection, mice were randomly divided into four groups (*n* = 7 mice per group). The CM (150 μL) was injected intramuscularly into the gracilis muscle of the medial thigh for four consecutive days. The physiological status of the ischemic limbs was monitored for 4 weeks after model establishment. A group treated with PBS (150 μL per limb) was used as the negative control. All animal experiments were performed in accordance with the guidelines of the Animal Welfare Act and the Guide for the Care and Use of Laboratory Animals, following protocols approved by the Institutional Animal Care and Use Committee (Sungkyunkwan University School of Medicine SKKUIACUC2020-06-11-1). All mice used in the experiments were housed under specific pathogen-free conditions.

### Histology and immunohistochemistry (IHC)

Ischemic limb muscles were retrieved 28 days after treatment. The tissues were fixed with 4% paraformaldehyde (Biosesang) in PBS, embedded in OCT compound (Scigen Scientific), frozen, and cut into 10-μm sections at −23 °C. After all samples were cut completely, the sections were subjected to immunofluorescent staining using anti-CD31 (1:50, Abcam, ab28364) and anti-smooth muscle (SM) α-actin (1:100, Abcam, ab5694) antibodies. Fluorescein isothiocyanate-conjugated secondary antibodies (1:50, Jackson ImmunoResearch Laboratories, 111-095-144) were used to visualize signals. The sections were counterstained with DAPI (Vector Laboratories) and examined under a fluorescence microscope (DFC 3000 G). Four images were randomly selected from each slide, and the fluorescent vessels were counted to quantify the CD31- and SM α-actin-positive vessels in the ischemic regions. Additionally, the sections were stained with H&E to examine muscle degeneration and tissue inflammation.

### Statistics and reproducibility

GraphPad Prism 7 software was used for all statistical analyses. In all the experiments, triplicate data were analyzed using a one-way analysis of variance with the Bonferroni test. Comparisons between two independent samples were performed using a two-tailed Student’s *t*-test. Results with *p* < 0.05 were considered statistically significant. The results are expressed as the mean ± standard deviation for all quantitative analyses.

### Reporting summary

Further information on research design is available in the Nature Research Reporting Summary linked to this article.

## Supplementary information

Supplementary Information

Description of Additional Supplementary Files

Supplementary Data 1

Reporting Summary

## Data Availability

Data is available upon request to the authors. All data generated or analysed during this study are included in this published article. Uncropped blot images are provided in Supplementary Fig. 5. All relevant data including the numerical and statistical source data that underlie the graphs in figures are provided in Supplementary Data 1.
