# Supplementary material for: Increasing angiogenic efficacy of conditioned medium using light stimulation of human adipose-derived stem cells
Source: Commun Biol. 2022 Sep 13;5:957. doi: 10.1038/s42003-022-03838-3 (PMC9470574; doi:10.1038/s42003-022-03838-3)
Supplement: Supplementary file 1 — Supplementary Information [file 42003_2022_3838_MOESM1_ESM.pdf]

Increasing angiogenic efficacy of conditioned medium using light stimulation of human adipose-derived stem cells

Yu-Jin Kim[1], Sang Ho Lee[1], Jisoo Im[1], Jihun Song[1], Han Young Kim[2,], Suk Ho Bhang[1,*]*

[1]School of Chemical Engineering, Sungkyunkwan University, Suwon 16419, Republic of Korea

[2]Department of Biomedical-Chemical Engineering, The Catholic University of Korea, Bucheon 14662, Gyeonggi, Republic of Korea.

***Co-corresponding author**

Suk Ho Bhang, Ph.D.

Email: sukhobhang@skku.edu; Tel.: +82-31-290-7242; Fax: +82-31-290-7272

Han Young Kim, Ph.D.

Email: hy0408@catholic.ac.kr; Tel.; +82-2-2164-4375; Fax: +82-2-2164-4375

Supplementary information

Supplemental Table 1. Primer sequences

Gene	Primer Sequence (5´-3´)
CASPASE-3	Forward: CCT GGT TAT TAT TCT TGG CGA AA
	Reverse: GCA CAA AGC GAC TGG ATG AA
HIF-1α	Forward: CAG TTA CGT TCC TTC GAT CAG TTG
	Reverse: TTT GAG GAC TTG CGC TTT CA
VEGF	Forward: GAG GGC AGA ATC ATC ACG AAG T
	Reverse: CAC CAG GGT CTC GAT TGG AT
BCL-2	Forward: CAA CAT CGC CCT GTG GAT GA
	Reverse: GGG CCA AAC TGA GCA GAG TC
FGF2	Forward: GAC GGC AGA GTT GAC GG
	Reverse: CTC TCT CTT CTG CTT GAA GTT
Hgf	Forward: GGC CCA CTC ATT TGT GAA C
	Reverse: CAT CCA CGA CCA GGA AC
Pcna	Forward: GCA CGT ATA TGC CGA GAC CT
	Reverse: CCG CCT CCT CTT CTT TAT CC
MyoD	Forward: ATC CGC TAC ATC GAA GGT CT
	Reverse: CGC TGT AAT CCA TCA TGC CA
MyoG	Forward: CAG TGA ATG CAA CTC CCA CA
	Reverse: CGA GCA AAT GAT CTC CTG GG

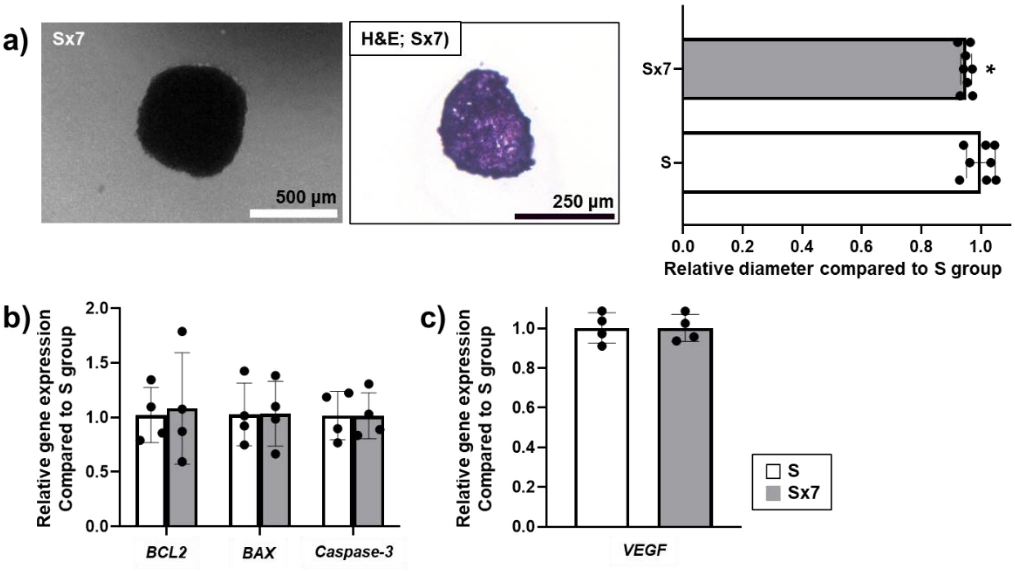

Supplemental Figure 1. Structural properties, cell viability, and angiogeic ability of densely-incubated hADSC spheroids. a) Representative morphology and H&E staining of the interior of densely-incubated hADSC spheroids. The relative diameter was compared with that of the S group as a control (n = 8, *p < 0.05 vs. S group). **b)** Cell viability of the densely-incubated hADSC spheroids was evaluated via the relative expression of *BCL-2*, *BAX,* and *CASPASE-3* in hADSCs using the S group as a control (n = 4). **c)** Relative expression of *VEGF* in densely-incubated hADSC spheroids using the S group as a control (n = 4). Data are presented as mean ± S.D.

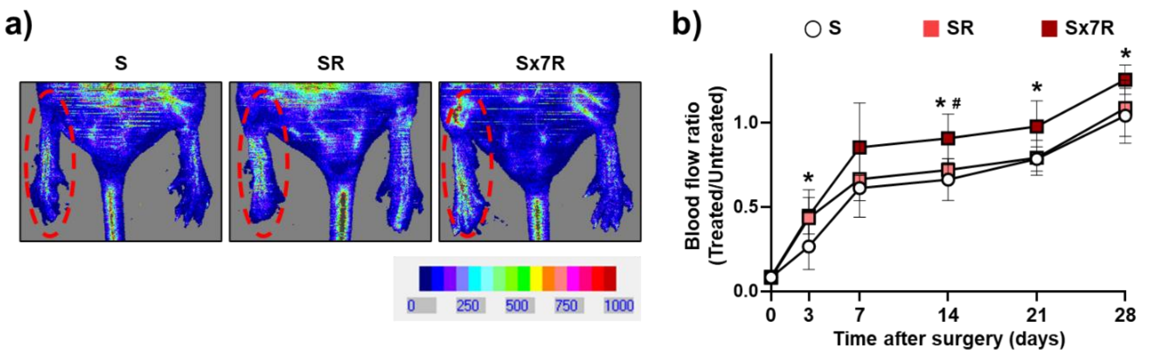

Supplemental Figure 2. a) Representative laser doppler imaging in each group at 28 days after the various treatments. **b**) Limb perfusions at 0, 3, 7, 14, 21, and 28 days after the various treatments (n = 5, *p < 0.05 vs. S group, #p < 0.05 vs. SR group). Data are presented as mean ± S.D.

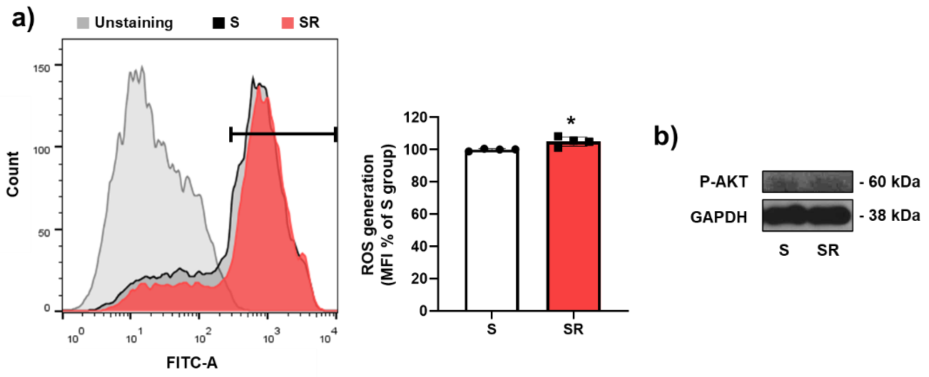

Supplemental Figure 3. a) Staining of intracellular ROS using DCF-DA (green), and its quantification expressed as a percent of the mean fluorescence intensity (MFI). The S group served as a control (n = 4, *p < 0.05 versus S group). **b**) Western blot analyses of P-AKT in hADSC spheroids treated with red light. Data are presented as mean ± S.D.

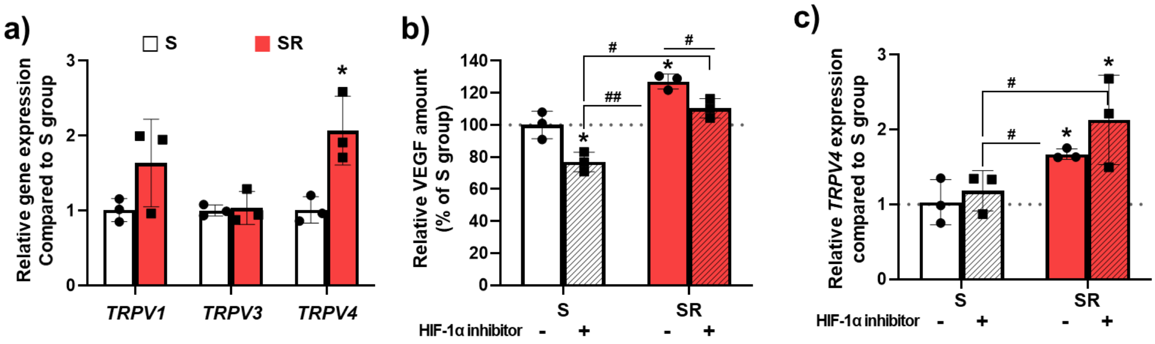

Supplemental Figure 4. **a**) Relative expression of *TRPV1*, *TRPV3*, and *TRPV4* in hADSC spheroids treated with red light (n = 3, *p < 0.05 vs. S group). **b**) The concentration of VEGF in the CM derived from S or SR with or without HIF-1α inhibitor (CAY10585), as evaluated using ELISA (n = 3, *p < 0.05 vs. S group without HIF-1α inhibitor, #p < 0.05 vs. each group, ##p < 0.001 vs. each group). **c**) Relative expression of *TRPV4* in the S or SR with or without HIF-1α inhibitor (n = 3, *p < 0.05 versus S group without HIF-1α inhibitor, #p < 0.05 versus each group). Data are presented as mean ± S.D.

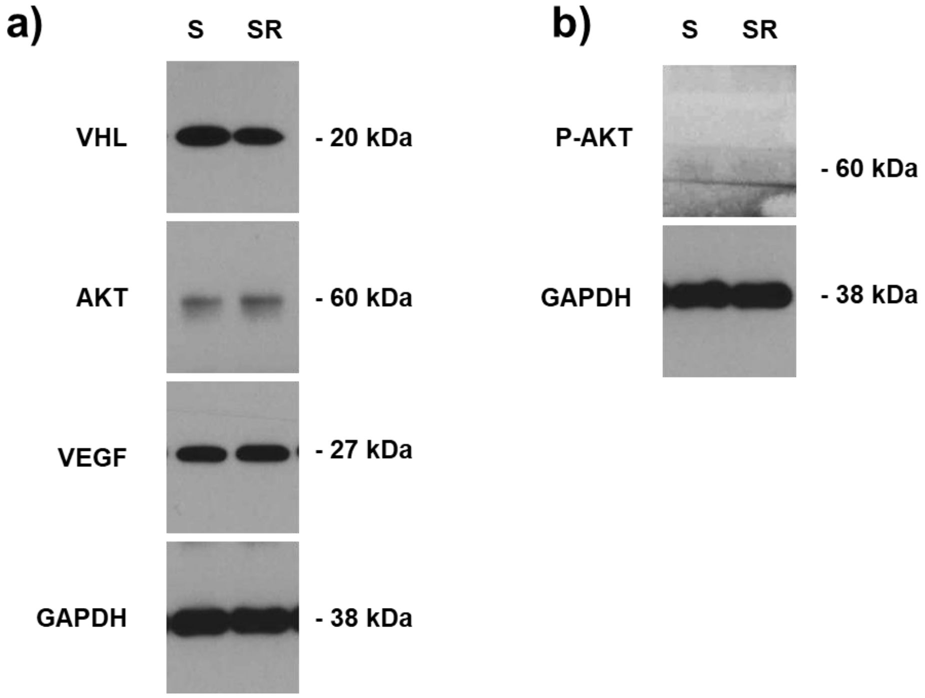

Supplementary Figure 5. a) Uncropped blot corresponding to Figure 2b. **b**) Uncropped blot corresponding to supplementary figure 3b.

Supplemental Notes 1. Laser doppler imaging analysis

A laser Doppler perfusion imager (Moor Instruments, Devon, UK) was used for serial non-invasive physiological evaluations of neovascularization. The mice were monitored by serial scanning of surface blood flow in the hindlimbs 0, 3, 7, 14, 21, and 28 days after treatment. Digital color-coded images were scanned and analyzed to quantify blood flow in ischemic regions from the knee joint to the toe. The mean values of perfusion were subsequently calculated.
